# Clinical outcomes of paliperidone long-acting injection in patients with schizophrenia: a 1-year retrospective cohort study

**DOI:** 10.1186/s12888-021-03513-7

**Published:** 2021-10-15

**Authors:** Hsue-Wei Chan, Chin-Yu Huang, Yung-Chieh Yen

**Affiliations:** 1 Department of Psychiatry, E-Da Hospital, 1 Yi-Da Road, Yan-Chau District, Kaohsiung, 824 Taiwan; 2 School of Medicine, I-Shou University, Kaohsiung, Taiwan

**Keywords:** Schizophrenia, Long-acting injectable antipsychotics, Hospitalization incidence, Emergency service, Paliperidone, Mirror study

## Abstract

**Background:**

Schizophrenia is a severe psychiatric disorder. Poor medical adherence increases relapse rate. Long-acting injection of antipsychotic agent is developed for improving medical adherence. In this study, we examined the effect of paliperidone long-acting injection (PLAI) treatment in patients with schizophrenia in a real-world setting.

**Methods:**

In this retrospective cohort study, 467 patients with schizophrenia were enrolled, treated with risperidone PLAI or oral antipsychotics, and followed for 1 year. Concomitant medication, namely anticonvulsants, antidepressants, anxiolytics, sedatives or hypnotics, anticholinergics, and beta-blockers, were administered. Patients were classified into 2 groups: the LAI group (patients received LAI for treatment) and the NLAI group (patients taking only oral antipsychotics). The incidence of hospitalization, the length of hospitalization, and the incidence of emergency room visits were assessed.

**Results:**

The LAI group had a higher incidence of psychiatric acute ward admission (NLAI group = 4.8%; LAI = 30.3%) and emergency room visits (NLAI group = 7.3%; LAI group = 36.0%) before enrolment. During the one-year follow-up, the incidence of acute ward admission and emergency room visit did not differ in the NLAI group (*P* = .586 and .241) compared with before enrolment, whereas both incidences were significantly decreased in the LAI group (*P* < .0001 in both of them).

**Conclusions:**

PLAI reduces the incidence of admission and emergency room visits.

## Background

Schizophrenia, with a prevalence of approximately 1% worldwide [1], is a severe psychiatric disorder with positive symptoms (eg, hallucinations, delusions, disorganized speech or behaviors, agitation, and aggression) related to psychosis and negative symptoms (eg, flat affect, social withdrawal, and cognitive dysfunction) [2, 3].

Auditory hallucination, which was reported to more frequently consist of negative voice content [4], causes distress, anxiety, depression, and behavioral disturbance [5]. Command auditory hallucinations that encourage harm to the self or others, sometimes in disturbing detail, can be controlling and very distressing to patients [6–9]. Furthermore, command hallucinations can increase the risk that patients will harm themselves or others [10–15]. Delusional thought content may be associated with irritability, depression, violence, suicide, and other risky behaviors [15–19]. Aggressive behaviors are influenced not only by psychosis, but also comorbid substance use or personality disorder [20]. Negative symptoms include affective flattening, alogia, avolition, anhedonia, and asociality [21]. Deficits in emotional expression and volitions are a distinct aspect of negative symptoms [22], and avolition is a critical component affecting patients’ functional outcomes [23].

Numerous types of antipsychotic agents have been developed for treating schizophrenia. These agents have been categorized into “first-generation antipsychotics” and “second-generation antipsychotics,” which mainly act by reducing the dopaminergic tone, thereby alleviating positive symptoms [24]. The second-generation antipsychotic agents are associated with a lower risk of developing extrapyramidal symptoms compared with first-generation antipsychotics. Furthermore, a literature review revealed that second-generation antipsychotic agents display neuroprotective effects [25].

Although antipsychotic agents may be effective in treating schizophrenia, their long-term therapeutic effects depend on medical adherence. Insight is an important predictor of medication adherence [26]. High medical adherence improves psychosocial functioning and increases symptom remittance [27]. Poor insight is a common feature among patients with schizophrenia; a study demonstrated that over 50% of patients with schizophrenia partially or completely lacked insight into their mental disorder [28–30]. A lack of insight is linked to poor medication adherence and poor prognosis [28, 31, 32]. Nonadherence to antipsychotic treatment is a critical factor in relapse [33]. The relapse of psychotic symptoms not only affects the patient’s daily life but also the paranoid ideation is associated with violence [34], which affects the patient’s and surrounding people’s safety.

Poor medical adherence also increases the relapse rate. The lack of awareness of this illness among patients, families, and the public can delay the detection of relapse symptoms, thus delaying timely intervention [35]. Repetitive relapses have negative consequences, such as functional decline, poor prognosis, poor future treatment response, and high health care cost [36]. Chronic symptoms affect the patient’s quality of life and impose considerable emotional, psychological, physical, time, and economic burdens on the patient’s caregivers [37, 38].

Long-acting injection (LAI) antipsychotic agents were developed as a treatment strategy for improving medication adherence. Several factors, including a patient’s character, history, relapse severity, cognitive problems, persistent aggressive behavior, medical access, and medical costs, are also considered when considering LAI antipsychotic agents [39, 40].

Studies have revealed that LAI is associated with a decreased mortality rate in patients with schizophrenia, which is even lower with second-generation LAIs [41]. A meta-analysis revealed that the rate of severe adverse effects did not differ between LAIs and oral antipsychotics [42].

Paliperidone palmitate LAI (PLAI) is a second-generation LAI antipsychotic agent with high efficacy in reducing disease severity and preventing relapse [43]. The efficacy of PLAI was demonstrated to be similar to that of first-generation LAIs, such as haloperidol LAI, but with less movement disorder [44]. Treatment with PLAI for schizophrenia was associated with a significantly longer time to relapse compared with oral antipsychotic agents [45]. The once-monthly PLAI was demonstrated to be beneficial in treating patients with schizophrenia comorbid with substance abuse [46]. In patients with schizoaffective disorder, the use of once-monthly PLAI improved all domain scores on the Personal and Social Performance scale [47].

In this study, we investigated the effects of PLAI in the real-world treatment of patients with schizophrenia. We recruited patients with schizophrenia who were receiving oral antipsychotic agents or PLAI treatment and compared their emergency room visit and rehospitalization rates.

## Methods

### Study design

This study was approved by the Institutional Review Board of E-Da Hospital. Data were obtained from a retrospective cohort study conducted during September 2014 and September 2018. A total of 467 patients with schizophrenia receiving different types of antipsychotic treatment were included in this retrospective cohort study. Among these patients, 178 received at least one PLAI injection, and 289 were treated with oral-form antipsychotics alone. Patients were thus separated into 2 groups: the NLAI group and the LAI group. The NLAI group consisted of patients receiving oral antipsychotic agents, whereas the LAI group consisted of patients receiving PLAI.

This observational study was conducted in a university hospital. Patients received psychiatric treatment in the outpatient department, with the emergency room and psychiatric ward available when necessary. This study determined clinical outcomes in patients with schizophrenia and the relationship with different pharmacological treatments during 1 year of follow-up.

The index date of the LAI group was defined by the time when a patient received the first dose of long-acting injectable antipsychotics; we then followed up the patient for 1 year post treatment and also traced back for 1 year prior the first injection to determine clinical outcome. For the NLAI group, the index date would be the time of enrollment.

### Inclusion and exclusion criteria

Male and female patients were included. Patients aged 18 years or more were recruited at the regional E-Da Hospital in Kaohsiung City, Taiwan, where they received psychiatric treatment. All patients were diagnosed as having schizophrenia according to the DSM-5 criteria.

Patients with severe medical conditions, pregnant or breast-feeding patients, and patients who did not regularly receive follow-up were excluded.

### Clinical outcomes

Patients’ demographic data, history of schizophrenia treatment, and data related to pharmacological treatment were retrospectively collected from hospital medical records. During the 1-year follow-up, the concomitant medication, hospitalization incidence, length of hospital stay, and emergency room visits were recorded.

### Treatment options

The recruited patients mainly received treatment in outpatient care. The antipsychotic selection and dosage were determined based on each patient’s clinical condition and psychiatric evaluation. Patients receiving PLAI were administered the injection during regular outpatient-department follow-ups.

### Statistical analysis

Patients were categorized based on the antipsychotic agents they received. Demographic data, baseline characteristics, and clinical outcomes were compared between the groups. The t test was used to compare the means of continuous variables between the groups; Fisher’s exact test was used to compare the distribution of categorical variable (sex) between the groups. In both the groups, the change between the hospitalization incidence before recruitment and the rehospitalization incidence after enrolment was examined using Fisher’s exact test.

## Results

From September 2014 to September 2018, 467 eligible patients with schizophrenia received treatment at E-Da Hospital. Of these 467 patients, 178 received PLAI, whereas 289 were treated with oral antipsychotic agents. These patients were followed for another year (until August 2019). These 467 patients were divided into 2 groups: the NLAI group and the LAI group. The NLAI group included patients with schizophrenia receiving oral antipsychotic agents, and the LAI group included patients with schizophrenia receiving PLAI.

The NLAI group comprised 156 men and 133 women with a mean age of 45.6 years, and the LAI group comprised 100 men and 78 women with a mean age of 40.4 years. Significant differences were observed in the mean age (*P* = .001), with no significant difference in sex (*P* = .702), between the groups (Table 1).

**Table 1:** Subject characteristics and mental health service utilization by treatment group

	NLAI (***N*** = 289)		*p*^*a*^	LAI (***N*** = 178)		*p*^*b*^	*p*^*c*^
	Before	After		Before	After		
Age, mean year (SD)	45.6 (16.2)			40.4 (11.9)			0.0001
Men, n (%)	156 (54)			100 (56)			0.702
Hospitalization, n (%)	14 (4.8)	18 (6.2)	0.586	54 (30.3)	21 (11.8)	< 0.0001	
Length of stay, mean days (SD)	35 (10.5)	37 (12.2)	0.069	33 (28.8)	35 (19.8)	0.890	
Emergency room visit, n (%)	21 (7.3)	30 (10.4)	0.241	64 (36.0)	14 (7.9)	< 0.0001	

Fisher exact test was used to compare differences for categorical variables; t-test was used to compare differences for continuous variables. LAI, patients receiving a long-acting injection of paliperidone; NLAI, patients receiving only oral antipsychotic

^a^differences before and after treatment in the NLAI group

^b^differences before and after treatment in the LAI group

^c^differences between the NLAI and LAI groups

Oral antipsychotic agents, sedatives or hypnotics, anticonvulsants, antidepressants, anxiolytics, anticholinergics, and beta-blockers were used as concomitant medication in both the groups. Theincidences of use of oral antipsychotics, sedatives or hypnotics, antidepressants, and anticholinergics were significantly higher in the NLAI group, with *P* values of <.0001, <.0001, .0002, and .00395, respectively. The incidence of anxiolytic agent use was higher in the LAI group with *P* < .0001. In the LAI group, patients received between 1 and 25 doses of PLAI, with a mean of 9.1 doses and a median of 10 doses during follow-up (Table 2).

**Table 2:** Concomitant medications by group of treatment

	NLAI (***N*** = 289)	LAI (***N*** = 178)	*p*
Oral antipsychotics (%)	269 (93.1)	28 (15.9)	< 0.0001
Risperidone	77 (28.6)	1 (3.6)	
Paliperidone	69 (25.6)	13 (46.4)	
Quetiapine	40 (14.9)	11 (39.3)	
Olanzapine	38 (14.1)	3 (10.7)	
Aripiprazole	15 (5.6)		
Amisulpride	12 (4.5)		
Ziprasidone	8 (3.0)		
Clozapine	5 (1.9)		
Zotepine	3 (1.1)		
Lurasidone	2 (0.7)		
Sedatives/hypnotics (%)	156 (54.0)	39 (22.2)	< 0.0001
Anticonvulsant (%)	93 (32.2)	60 (34.1)	0.761
Antidepressant (%)	98 (33.9)	32 (18.2)	0.0002
Anxiolytics (%)	23 (8.0)	67 (38.1)	< 0.0001
Anticholinergics (%)	56 (19.4)	21 (11.9)	0.00395
β-blocker (%)	26 (9.0)	20 (11.2)	0.429

Fisher exact test was used to compare differences between groups

**Fig. 1:**
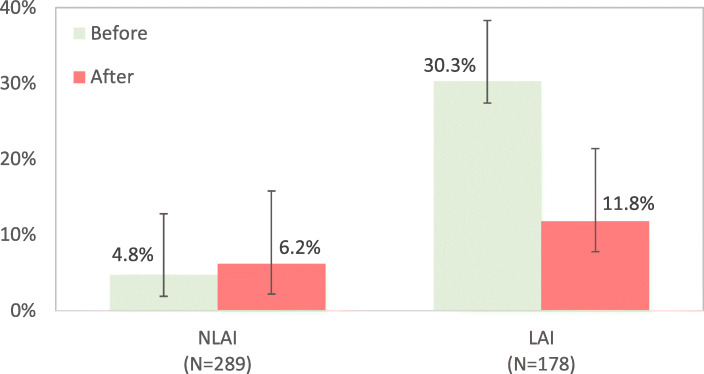
Hospitalization and rehospitalization by treatment group

**Fig. 2:**
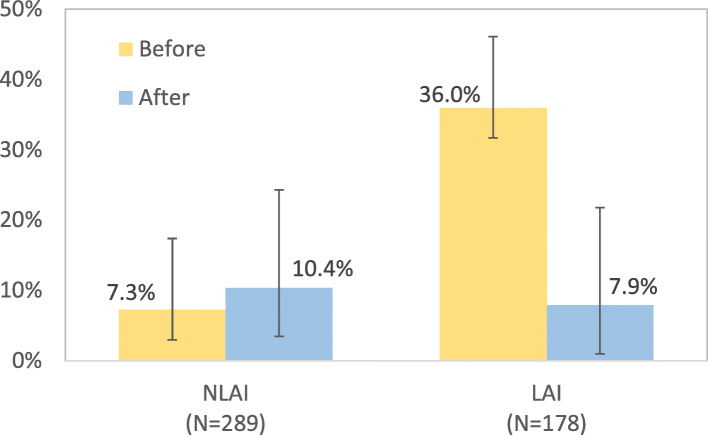
Emergency room utilization by treatment group

Acute psychiatric ward hospitalizations, hospital stay durations, and emergency room visits before enrolment were measured in both the NLAI and LAI groups. The LAI group had a higher incidence of psychiatric acute ward admission before enrolment (NLAI group, 4.8% [95% CI 2.9 to 8.0%]; LAI group, 30.3% [95% CI 23.6 to 37.1%]). The length of hospital stay was similar in the 2 groups (NLAI group, 35 days; LAI group, 33 days). The incidence of emergency room visits was higher in the LAI group (NLAI group, 7.3% [95% CI 4.3 to 10.1%]; LAI group, 36.0% [95% CI 28.9 to 43.0%]; Table 1, Figs. 1 and 2).

During the one-year follow-up, the acute ward admission incidence was 6.2% (95% CI 4.0 to 9.6%0 in the NLAI group and 11.8% (95% CI 7.1 to 16.5%) in the LAI group; the mean hospital stay duration was 37 days in the NLAI group and 35 days in the LAI group; the incidence of emergency room visits was 10.4% (95% CI 6.9 to 13.9%) in the NLAI group and 7.9% (95% CI 3.9 to 11.8%) in the LAI group. The admission incidence was not significantly different before and after enrolment in the NLAI group (*P* = .586), whereas it was significantly decreased in the LAI group (*P* < .0001); the length of hospital stay did not differ significantly between before and after enrolment in either group (*P* = .069 and .890 in NLAI and LAI groups, respectively); the incidence of emergency room visits did not differ significantly between before and after enrolment in the NLAI group (*P* = .241), whereas it was significantly decreased after enrolment in the LAI group (*P* < .0001; Table 1, Figs. 1 and 2).

## Discussion

LAIs of antipsychotic agents have been demonstrated to be effective in treating patients with schizophrenia. A review article suggested certain considerations when prescribing LAIs of antipsychotic agents, such as nonadherence, the severity of episode or relapse, cognitive problems, memory problems, substance misuse, social support level, loss of function risk, medical access, cost, patient autonomy, and fear of needles and pain [39].

LAIs of antipsychotic agents are undoubtedly one of the most effective treatment choices, with the advantage over the oral antipsychotic agents of continuous coverage of the medication effect.

When poor medication adherence, the severity of the episode, and the severity of relapse are considered, LAIs of antipsychotic agents may be a treatment of choice for this group of patients because poor adherence increases the risk of disease relapse. A study revealed that the use of PLAI in combination with clozapine is effective in treating patients with treatment-resistant schizophrenia [48]. Our study demonstrated that the year before enrolment, the LAI group had higher incidences of acute psychiatric ward admission and a higher number of emergency room visits than did the NLAI group. However, 1 year after enrolment, the incidence of admission and the number of emergency room visits both significantly decreased in the LAI group, but they did not significantly differ from those in the NLAI group. These findings indicate that the use of PLAI reduced disease severity in patients with severe disease. The range of doses of PLAI in the LAI group was 1 to 25, with a mean of 9.1, which suggests that PLAI was still effective in reducing disease severity in patients who did not receive PLAI treatment regularly, such as 1–9 doses a year.

Studies have revealed that paliperidone is effective in the treatment of schizoaffective disorder [49, 50]. The once-monthly PLAI used to treat patients with acute exacerbation of schizoaffective disorder alleviated psychotic symptoms and manic and depressive symptoms [49]. Paliperidone has also been demonstrated to alleviate depressive symptoms in patients with schizophrenia [51]. In our study, the LAI group used fewer concomitant medications than did the NLAI group, including antidepressants, which suggests that PLAI can also treat depressive symptoms in patients with schizophrenia.

Several mirror-image studies have been conducted to examine the effect of PLAI. A study in London [52] enrolled 173 patients (70% patients with schizophrenia and 30% with schizoaffective disorder, bipolar disorder, or other diagnoses) and followed their condition progression for 3 years after initial PLAI injection. Furthermore, their data were retrospectively reviewed for 3 years before enrolment. The result revealed that full compliance patients had decreased hospital admissions and bed days. A multicenter mirror study in Turkey [53] revealed that the use of PLAI reduced the incidences of hospital visits because of relapse and the median number of hospitalizations. The Positive and Negative Symptom Scale score decreased over the course of PLAI treatment. A 2-year study in Korea [54] enrolled patients who received at least 2 dosages of PLAI and demonstrated that the number of admissions and the total number of bed days were both reduced after PLAI administration. Cohort study also revealed the effect of PLAI in lowering risk of psychiatric rehospitalization [55]. These results accord with our finding that the use of PLAI reduced the incidence of hospitalization.

We further determined that the incidence of emergency room visits decreased after PLAI treatment. Therefore, the use of PLAI appears to reduce disease severity in patients with schizophrenia.

There were rare mirror image studies of PLAI, especially in Asia. Two studies carried out in Korea [54, 56] enrolled only patients receiving PLAI treatment to compare the difference of long-acting injectable antipsychotic treatment and prior oral antipsychotic treatment. In our study, we enrolled not only patients receiving PLAI, but also patients receiving oral antipsychotic agent in the same hospital at the same period of time. Therefore, we can compare the differences between these 2 groups of patients including the patient characters and their outcome. As revealed in the result, the LAI group had higher hospitalization incidence and emergency room visit incidence than NLAI group 1 year before the enrollment. This implied that the LAI group was composed of a group of patients with higher disease severity than the NLAI group. After 1 year of treatment, the hospitalization incidence and emergency room visit incidence both significantly dropped in LAI group to about the same level of NLAI group. The findings of this study add more evidence on the effectiveness of PLAIs for schizophrenia and hence important for clinicians.

In this research, we found that psychiatrist tended to use long-acting injection antipsychotic agent for treating severe patients, and the using of long acting injection of antipsychotic agent did significantly improved disease severity. In Taiwan, the long-acting injection of antipsychotic agent was still not that commonly used in treating patients in comparison to the Western countries. We hoped that this result could be a reference for psychiatrist to consider the long-acting injection of antipsychotic agent as an effective treatment of choice while treating patients with schizophrenia.

Some limitations of this study should also be considered. As a non-interventional study, we did not use propensity score matching for relevant clinical characteristics. The 2 groups of patients (receiving PLAI treatment and oral antipsychotic agent treatment) might be different in their nature, however, in this observation study, we could only use the hospitalization incidence before enrolment to demonstrate the difference in character of these 2 groups of patients. Since the mechanism of psychosis deterioration could be complicated, the patient’s nature such as vulnerability to psychosis, multiple psychosocial stressors (such as economical stress), medical adherence and the interaction between these factors contribute the relapse or recurrence of psychosis. Since data were retrieved from medical records, it would be difficult to clarify and measure the weighting of influences of these factors on each of the patients.

## Conclusions

This study demonstrates PLAI reduces the incidence of the admission incidence and emergency room visits of patients with schizophrenia.

## Data Availability

All data generated or analyzed during this study are included in this published article.

## Abbreviations

PLAI: Paliperidone long-acting injection
LAI: Long-acting injection
